# Whole body bone scintigraphy in osseous hydatosis: a case report

**DOI:** 10.1186/1752-1947-1-93

**Published:** 2007-09-19

**Authors:** Abdolali Ebrahimi, Majid Assadi, Mohsen Saghari, Mohammad Eftekhari, Amir Gholami, Reza Ghasemikhah, Sakineh Assadi

**Affiliations:** 1 Department of Oncology and Nuclear Medicine, The Persian Gulf Health Research Center, Bushehr University of Medical Sciences, Bushehr, Iran; 2 Research Institute for Nuclear Medicine, Shariati Hospital, Tehran University of Medical Sciences, Tehran, Iran; 3 Department of Parasitology, Faculty of Health Sciences, Tehran University of Medical Sciences, Tehran, Iran

## Abstract

Hydatid disease is common in many parts of the world, and causes considerable health and economic loss. This disease may develop in almost any part of the body.

Bone involvement is often asymptomatic, and its diagnosis is primarily based on radiographic findings. A whole body bone scan is able to show the extent and distribution of lesions.

We describe an unusual case of multifocal skeletal hydatosis and also explain the clinical and diagnostic points. We hope to stimulate a high index of suspicion among clinicians to facilitate early diagnosis and to consider this disease as a differential diagnosis in cases of multiple abnormal activity in bone scintigraphy especially among people in endemic areas.

## Background

**Figure 1:**
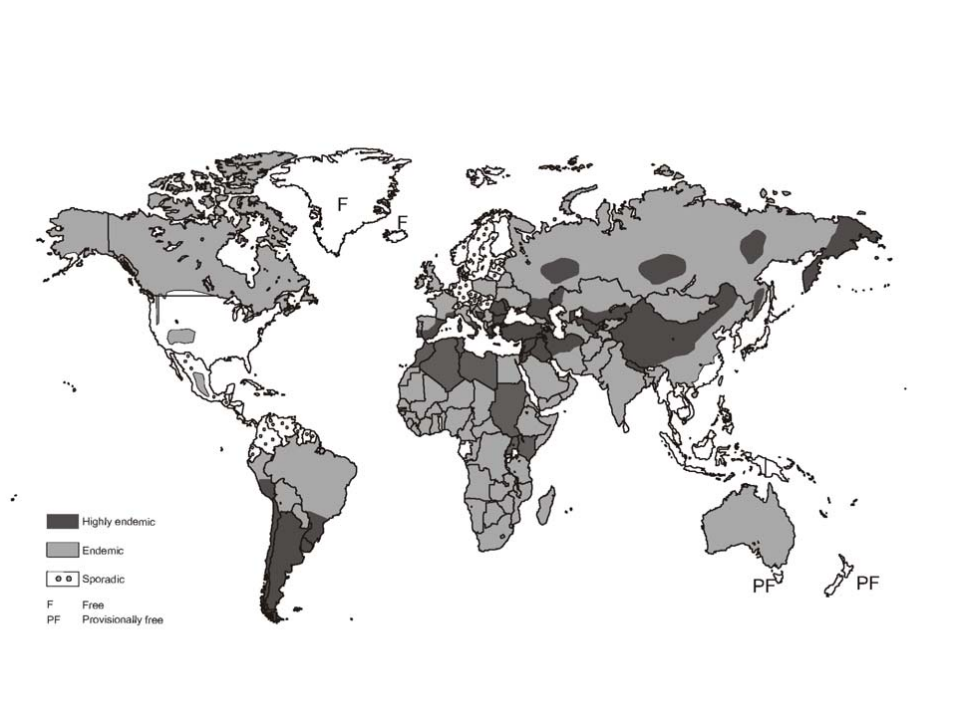
Approximate geographic distribution of Echinococcus granulosus [15].

Echinococcosis is a zoonotic infection caused by adult or larval (metacestode) stages of cestodes belonging to the genus Echinococcus and the family Taeniidae. The adult Echinococcus lives in the small intestine of carnivores such as dogs, wolves, jackals and the excreted eggs of the worm are scattered through the stool of these animals (Schantz, 1991; Thompson, 1995). Echinococcus granulosus has a worldwide geographic distribution and occurs in all continents including circumpolar, temperate, subtropical and tropical zones [1]. The highest prevalence of the parasite is found in parts of Eurasia, Africa, Australia and South America (Figure 1). Within endemic zones, the prevalence of the parasite varies from sporadic to high, but only a few countries can be regarded as being free of E. granulosus (Figure 1).

Hydatid disease may develop in almost any part of the body. Most hydatid cysts occur in the liver (59–75%), or in the lung (27%). Involvement of the kidney (3%) or brain (1–2%) is rare [2].

Bone localization is also rare comprising 0.5% to 2.5% of all human hydatidosis [3]. The diagnosis of osseous hydatidosis is primarily based on radiological findings. Treatment is difficult and recurrence is common [4].

Therefore, plain radiography, CT scan, and MR imaging are helpful in diagnosing skeletal cystic echinococcosis [5]. Osseous foci may manifest as bone pain and deformity, particularly among patients in the 30–60 year olds age group [6,7]. Hereby, we present an unusual case of multifocal skeletal hydatosis and also explain the clinical and diagnostic points to facilitate early diagnosis of this condition.

## Case presentation

The patient was a 53 year old man with a history of osseous hydatosis since the age of 30. He had had three surgical operations due to the disease. He had low back pain and radicular pain in both legs which had been aggravated over the past month. His past history for any other illnesses was negative. There wasn't a history of coughing or other respiratory symptoms. His general condition was good. On examination we detected low back tenderness in the lumbar area. He had a painful tumor-like mass in the right hemipelvis which was slightly warm without overlying erythema. Straight leg raising test (SLR) was positive in both legs. He had motor and sensory deficit, muscular atrophy and gate disturbance. No other abnormal findings were detected.

**Figure 2:**
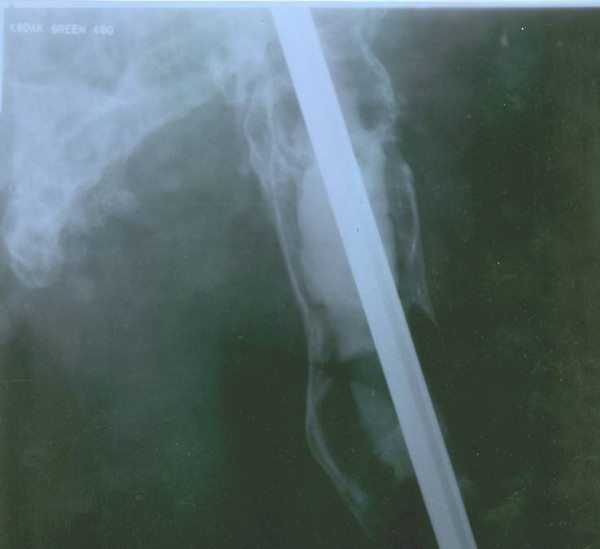
Plain x-ray of right femur showed advanced lytic destruction of bone, centered at the proximal two thirds of the right femur. There is also a nailing due to previous surgery.

**Figure 3:**
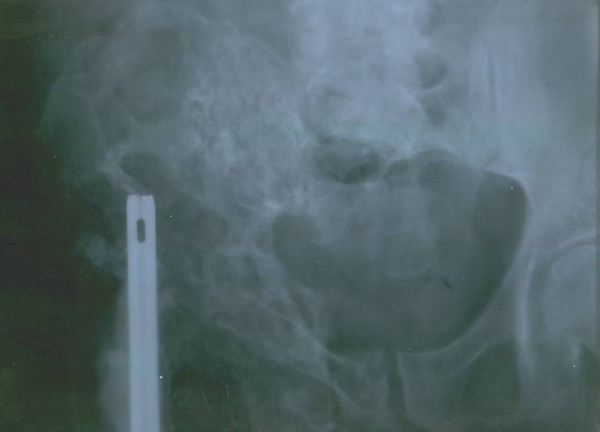
Plain x ray of the pelvis revealed marked lytic and sclerotic lesions involving the right hemipelvis.

**Figure 4:**
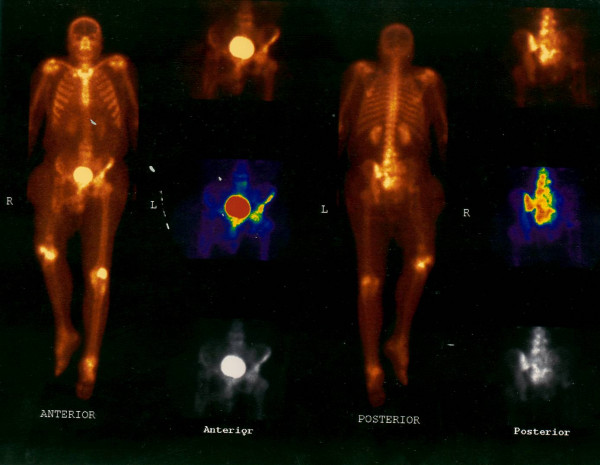
Whole body bone Scintigram showed multiple foci of increased radiotracer uptake in the lower lumbar spine, the left sacroiliac region and the right knee. In addition there was soft tissue bulging in the right hemipelvis as well as a displaced and disconfigured right femur.

Simple x-ray of the right femur showed advanced lytic bone destruction, centered in the proximal two thirds of the right femur. In addition there were the appearances of nailing due to previous surgery (fig. 2). The plain pelvic x-ray also revealed marked lytic and sclerotic lesions involving the right hemipelvis (Figure 3). A whole body bone scintigram revealed multiple foci of increased radiotracer uptake in the lower lumbar spine, left sacroiliac region and right knee. In addition, there was soft tissue bulging in the right hemipelvis as well as a displaced and disconfigured right femur (Figure 4). Chest x ray was normal.

## Conclusion

Hydatidosis is a common disease of both humans and animals resulting from infection with the larval stage of the *Echinococcus granulosus *tapeworm. This disease is common in many parts of the world and causes considerable health and economic loss [8].

Liver and lungs are the most commonly involved organs and involvement of bone and muscles is uncommon [9]. The prevalence of bone infection is 1.1% in nonendemic to 4% in endemic areas [10]. In descending order, the vertebrae, long bone epiphyses, pelvis, skull and ribs are most frequently affected. Soft tissue involvement with calcification is highly suggestive of this disease [11].

Men are involved more than women [9] and the peak age of disease is 21–40 years [10]. Patients with spinal column hydatid cyst may experience lumbar pain, paresthesia, paraparesia and even paraplegia or sphincter dysfunction. Neurological symptoms of this disease may be due to spinal involvement and compression effects on the spinal cord, or direct cord involvement [10].

Although the incidence of hydatid disease has decreased as a result of education and control measures, there are still foci of concern in South America and sporadic cases still occur in the United States, Europe, the Middle East, and Asia [12]. Hydatid cysts may lie dormant in the bone for as long as 40 years and most skeletal hydatid cysts have been detected in adults. Skeletal cystic echinococcosis lesions may be single or multiple [5].

A lucent expansile lesion with cortical thinning is the most frequent radiological pattern, and pathological fractures are common [7].

As hydatid disease of bone remains asymptomatic over a long period, it is usually detected after a pathological fracture or secondary infection or following the onset of compressive myelopathy in the case of vertebral lesions [7].

The differential diagnosis of skeletal cystic echinococcosis includes other infectious lesions (e.g. tuberculosis), fibrous dysplasia and tumors (including simple bone cyst, aneurismal bone cyst, plasmocytoma, osteosarcoma, chondorsarcoma, chondromyxoid fibroma, lymphoma, giant cell tumors, brown tumor and metastases). Diagnosis is primarily based on findings of X-ray and CT scans [5,7]. Whole body bone scintigraphy (WBBS) is able to show the extent and distribution of lesions. WBBS has a high sensitivity but has a poor specificity for osteopathological lesions.

Immunodiagnostic procedures for serum antibody detection such as enzyme-linked immunosorbent assay (IgG-ELISA), the indirect hemagglutination antibody test (IHAT), the latex agglutination test (LAT), the immunofluorescence antibody test (IFAT), immunoelectrophoresis (IEP) and some other tests are used for the etiological confirmation of imaging structures suggestive for cystic echinococcosis or for diagnosis or differential diagnosis in cases of uncharacteristic imaging findings [13].

The initial location of the lesion in long bones is metaphyseal or epiphyseal, later extending to the diaphysis. Potential complications include pathological fracture, infection, and fistulisation of the abscess [7].

Surgery is the treatment of choice for hydatid bone lesions. Many authors have advocated wide resection of the involved bone along with the surrounding soft tissue as the only definitive treatment of the condition, with or without chemotherapy using albendazole or mebendazole [14].

Growth in the direction of least resistance, in time, causes cortical destruction with extension of the cyst into surrounding soft tissues. This condition is rarely encountered in childhood [7]. Hydatid bone disease should be considered in the differential diagnosis of osteolytic lesions, especially in endemic areas. The presence of a periosteal reaction, osteosclerosis, and calcification are not specific for hydatid bone disease [7].

Finally, hydatid bone disease should be considered in the differential diagnosis of osteolytic lesions in radiological imaging as well as single or multiple abnormal uptakes in the whole body bone scan especially among people living in endemic areas.

## Abbreviations

ELISA = enzyme-linked immunosorbent assay; IEP = immunoelectrophoresis; IFAT = immunofluorescence antibody test; IHAT = indirect hemagglutination antibody test; LAT = latex agglutination test; SLR = Straight leg raising test; WBBS = Whole body bone scintigraphy.

## Competing interests

The author(s) declare that they have no competing interests.

## Authors' contributions

AE revised the article for intellectual content details and helped to draft the manuscript .MA participated in writing of the manuscript and interpretation of the scintigraphic figures. MS, ME, AG, RG, SA, supervised the acquisition process and interpreted the scintigraphic and radiological images. All authors read and approved the final manuscript.

## Consent

Written informed consent was obtained from the patient for publication of this case report.
